# COVID-19 pandemic-related changes in wellness behavior among older Americans

**DOI:** 10.1186/s12889-021-10825-6

**Published:** 2021-04-19

**Authors:** Elgloria Harrison, Lillie Monroe-Lord, Andrew D. Carson, Anne Marie Jean-Baptiste, Janet Phoenix, Phronie Jackson, B. Michelle Harris, Elmira Asongwed, Matthew L. Richardson

**Affiliations:** 1 College of Agriculture, Urban Sustainability and Environmental Sciences, University of the District of Columbia, 4250 Connecticut Ave, NW, Washington, DC 20008 USA; 2 Lehman College City University of New York, 250 Bedford Park Boulevard West, Bronx, NY 10468 USA; 3 The George Washington University Milken Institute School of Public Health, 2175 ‘K’ Street NW, Suite 500, Washington, DC 20037 USA

**Keywords:** Coronavirus, Resilience, Quality-of-life, Food security, Physical activity, Nutrition, Senior citizens

## Abstract

**Background:**

COVID-19 has taken its toll on citizens in all 50 states of the United States. The United States (U.S.) leads the world with 30,291,863 confirmed reported cases and 549,664 deaths as of March 29, 2021 compared to globally confirmed cases at 127,442,926 and 2,787,915 deaths as of March 29, 2021. The U.S. federal government primarily left the response to the virus to individual states, and each implemented varying measures designed to protect health of citizens and the state’s economic well-being. Unintended consequences of the virus and measures to stop its spread may include decreased physical activity and exercise, shifting access and consumption of food, and lower quality-of-life. Therefore, our primary goal was to quantify the impact of COVID-19 on health and well-being by measuring changes in physical activity, mental health-quality of life, food security and nutrition in adults ages 40 and older. We believed shifts in health behaviors would be more prevalent in minorities, less educated, lower socio-economic status, older adults, and those with underlying health conditions, so a secondary goal was to determine the impact of COVID-19 on these sub-populations.

**Methods:**

We conducted an online survey with 9969 adults 40 years and older between 9 August and 15 September 2020 in urban areas across the four U.S. census regions. The survey included questions about demographic variables, pre-existing health conditions, physical activity, access to food, quality-of-life, and nutritional food status and asked participants to respond with information from pre-pandemic and pandemic conditions. We used paired-sample t-tests to detect changes in variables after the start of the pandemic and Cohen’s *d* to determine effect sizes.

**Results:**

Our main findings showed a decrease in physical activity since the onset of COVID-19 for minorities and non-minorities. Food security also slightly increased for minorities during the pandemic, but we found no other changes in food security, quality-of-life indicators, or nutritional status of those who responded to this survey.

**Conclusions:**

It is concerning that physical activity declined. Such activity helps maintain physical and mental health, and it is also an important time to socialize for many older adults. In many ways, our data indicate that the older adult population in U.S. cities may be more resilient than expected during the pandemic. However, the pandemic could have negative impacts that we did not detect, either due to the survey instrument or the timing of our survey, so the health and well-being of older adults should continue to be monitored in order to mitigate potential negative impacts.

## Background

Even though COVID-19 continues to be a global health emergency and the United States (U.S.) leads the world with 30,291,863 confirmed reported cases and 594,664 deaths as of March 29, 2021 compared to globally confirmed cases at 127,442,926 and 2,787,915 deaths as of March 29, 2021 [1]. The lack of a coordinated public health response from the federal government at the start of the pandemic in the U. S. was crippling and led to the virus spreading unabated [2]. Some states enacted measures that included stay-at-home policies, closing of businesses, face coverings, and social distancing, whereas other states did not [3, 4]. Unintended consequences of the virus and measures to stop its spread may include decreased physical activity and exercise, shifting consumption patterns and access to food, and feelings of anxiety [5–7].

### Physical activity

As COVID-19 continues unrelentingly, barriers to physical activity and exercise may be higher for some groups of people than others. For example, closure of gyms, bowling alleys, and supervised exercise classes, combined with a lack of home gym equipment, prevented a continuation of physical activity in older adults [8]. We defined well-being from a public health perspective as meeting physical and mental health approaches to disease prevention [9]. In addition to lost direct benefits from exercise, a reduction in the ability for older adults to socialize leads to an increased likelihood that they may suffer the loss of independence, which in turn may lead to cognitive impairment [10]. Those who can maintain higher levels of physical activities during the pandemic may display lower levels of depression and anxiety, which are key indicators of quality-of-life [11].

Whereas the ability to maintain an active lifestyle decreased during COVID-19, sedentary behaviors such as sitting, sleeping, and engaging in more television or computer watching increased [12].

 Mobility of citizens was restricted to essential workers and essential services in some local jurisdictions and states where stay-at-home policies were enacted to keep citizens safe.

[3, 4]. In New Zealand, which also restricted movement of citizens, a cross-sectional survey of 238 adults 18 years and older, between March–May 2020 found that 85% of the participants maintained regular physical activity prior to the lockdown, but only 49% were able to maintain regular exercise routine after the lockdown [13, 14]. Another study reported a decline in step count worldwide associated with confinement periods between January and March 2020 due to COVID-19 [15]. This research did not report the intensity of physical activity, but the authors believe that there is a negative linear relationship between decreases in step count and decreases in physical activity. In a health context, this decline in routine physical activity and exercise is concerning because these activities confer health benefits that mitigate risks associated with chronic diseases [6].

#### Quality-of-life (physical and mental health)

We used the Center for Disease Control’s definition of quality-of-life for individuals, which includes physical and mental perceptions (e.g., energy level, mood), health risks and conditions of functional status, social support, and socioeconomic status [16]. Recent research explored the relationships between resilience, hope preventive behaviors, subjective well-being, and psychological health among adults and found that high levels hope had a positive effect on resilience which translated to better mental well-being [17]; while another study reported anxiety measures of 600 participants using the Self-Rating Anxiety Scale where they compared anxiety risks they found that 93.67% had no anxiety symptoms, 6.33% felt anxiety, 82.83% had no symptoms of depression and 17.17% were depressed, women suggesting most participants were psychologically stable [18].

Individuals with high physical activity levels during the pandemic experienced lower levels of depression and anxiety and higher levels of quality-of-life [19]. Research examining effects of COVID-19 on mental health of older adults with a pre-existing major depressive disorder found that although older adults may experience physical distancing, they were not necessarily socially isolated: social isolation was mitigated for many by virtual connections with friends and family [10]. Even though physical activity was not included as a variable in the study, their findings suggest that physical activity could be introduced virtually as a source of social connection. Interestingly, another study looked at the physical activity levels of patients with cardiac disease in New York City and Minneapolis/Saint Paul and found a marked decline in physical activity due to pandemic restrictions that did not return to pre-pandemic levels even when restrictions began to ease [20]. Finally, social isolation induced by quarantine restrictions may cause mental health disturbances such as acute stress, depression, irritability, poor sleep habits, fear, and anxiety. Mental health stressors and poor sleep affect the immune system by decreasing the human body’s ability to fight infection [21, 22].

#### Food security and nutrition

Beyond access to physical activity and key social interactions that influence quality-of-life, COVID-19 restrictions may influence availability, access, and selection of nutritional food due, in part, to widespread closure of affordable food outlets [23]. Food security and nutritional intake has been used interchangeably to convey the necessary components of a food secure household; however, these terms are independent. Food security is defined as all people having access at all times to enough food for an active, healthy life [24]. The United States Department of Agriculture (USDA) defines ranges of food security as: 1) high food security – no food-access problems or limitations and 2) marginal food security one or two indicators of food access limitation [24]. The ranges for food insecurity are 1) low food security – indicators of food access limitations but no reduction in food intake and 2) very low food security – “disrupted eating patterns and reduced food intake” [24]. Nutritional intake refers to the quality of an individual’s diet to prevent malnutrition and improve body function. Nutritional intake should focus on maximizing consumption of fruits, vegetables, and healthy proteins to mitigate negative effects of noncommunicable diseases and complications such as high blood pressure, high cholesterol, obesity, and diabetes [25, 26].

Research supports physical activity and exercise as a non-pharmaceutical response to maintain good respiratory, cardiovascular, musculoskeletal, and mental well-being [11]. The CDC has suggested that the use of the term well-being is a valid population outcome measure [9]. Food security, nutritional food intake, and high quality-of-life increase resilience in older adults. Therefore, our primary goal was to quantify the impact of COVID-19 on physical and mental well-being by measuring changes in physical activity, physical and mental health (quality-of-life), food security, and nutrition in adults ages 40 and older. We predicted decreases in physical activity, food security, nutrition, and well-being associated with COVID-19. We believed these shifts in health behaviors would be more prevalent in minorities, less educated, with lower socio-economic status, older adults, and those with underlying health conditions, so a secondary goal was to determine the impact of COVID-19 on these sub-populations.

## Methods

### Participants and sampling

Our research is a cross-sectional study designed to determine the impact of COVID-19 on physical activity, food security, mental health (quality-of-life), and diet among vulnerable populations who live in urban cities and were more likely to experience severe symptoms or likely to die from complications associated with COVID-19. We sampled 9969 participants ages 40 years or older between 9 August through 15 September 2020. Relying on the prospective participant panel of individuals willing to complete online surveys, Qualtrics [27] managed the recruitment process using specifications provided by the authors with the goal of obtaining a sample from urban cities across the four census regions of the United States. Qualtrics sought to obtain a sample that was balanced by sex (male, female) and a combination of race (White, Asian, Black) and ethnicity (Hispanic, Non-Hispanic). Urban cities, as defined by the U.S. Census Bureau, have a population of more than 50,000 [28]. In addition to using the categories of race and ethnicity, a Minority Status construct was defined as “yes” if race was anything other than White (non-Hispanic), with minority status otherwise “no”.

### Measures

In addition to demographic questions, the survey included questions about pre-existing health conditions and a set of four constructs assessed under two conditions: “before” (thus, a retrospective condition) and “since” the start of COVID-19 in the U.S. Specifically, participants completed four subscales measuring these constructs in the “before” condition, and then participants completed the same set of four subscales in the “since” condition. These constructs included physical activity, food security, quality-of-life (physical and mental), and nutrition.

### Pre-existing health conditions

Participants selected options from a list of 19, including arthritis, back issues, fibromyalgia, hearing impairment, high cholesterol, hip issues, knee issues, macular degeneration, multiple sclerosis, osteoporosis, Parkinson’s disease, shoulder issues, stroke, visual impairments/eye health issues, lung disease (e.g., asthma, COPD, chronic bronchitis), cancer, kidney disease, heart attack, high blood pressure, and other (free response). We derived an overall index of health problems by adding the number of conditions, which yielded a maximum score of 20 (including the diabetes response). In addition, we developed an index of conditions (possible range 0–6) that have been linked to increased COVID-19 severity, which included lung disease, cancer, kidney disease, heart attack, high blood pressure, and diabetes [29].

### Physical activity

We assessed physical activity with a two-item scale adapted from the National Health Interview Survey [30] (see Table 1 for questions and scores assigned to responses), which has been a validated instrument since 1950 [31].Scores may range from 0 to 6, with higher scores indicating higher levels of physical activity. Physical activity was assessed by calculating separate scores for pre- and pandemic conditions and compared using a paired-sample t-test [32]. The two items making up this subscale had Pearson-r correlations of 0.49 and 0.52 for pre-pandemic and pandemic conditions, respectively (both significant at *p* < 0.05, *n* = 9669). The variable “physical activity change,” is the result of subtracting the pre-pandemic score from the pandemic score.

**Table 1:** Items contributing to the assessment of physical activity in adults ages 40–100 in the U.S

Question^a^	Points assigned^b^
In general, how would you describe your physical activity level?	No activity = 0 Low activity = 1 Somewhat low activity = 2 Somewhat high activity = 3 High activity = 4
In the past 3 months, have you participated in regular exercise (defined as: planned, structured, repetitive activity with an objective to improve or maintain physical fitness)?	No = 0 Yes = 2

^a^Questions adapted from the National Health Interview Survey [30]

^b^Scores for physical activity range from 0 to 6; higher scores indicate higher levels of physical activity

### Food security

We assessed food security through a four-item subset of the short form of the Household Food Security Scale [33, 34] (see Table 2 for questions and scores assigned to responses). The initial total score calculated for each participant was subtracted from eight so that a higher number indicated higher levels of food security. Scores may range from 0 to 8. Food security was assessed by calculating separate scores for pre- and pandemic conditions and compared with a paired sample t-test [32]. The internal consistency of the Food Security Scale was 0.88 and 0.92 for the pre-pandemic and pandemic conditions, respectively, indicating good reliability for the measure. The variable “food security change,” is the result of subtracting the pre-pandemic score from the pandemic score.

**Table 2:** Items contributing to the assessment of food security in adults ages 40–100 in the U.S

Question^a^	Points assigned^b^
The food that (I/we) bought just didn’t last, and (I/we) didn’t have money to get more. Was that often, sometimes, or never true for (you/your household) in the last 12 months?	Often true = 2 Sometimes true = 1 Never true = 0 Don’t know = 0
(I/we) couldn’t afford to eat balanced meals. Was that often, sometimes, or never true for (you/your household) in the last 12 months?	Often true = 2 Sometimes true = 1 Never true = 0 Don’t know = 0
In the last 12 months, did you ever eat less than you felt you should because there wasn’t enough money for food?	Yes = 2 No = 0 Don’t know = 0
In the last 12 months, were you ever hungry but didn’t eat because there wasn’t enough money for food?	Yes = 2 No = 0 Don’t know = 0

^a^Questions from the short form of the Houseful Food Security Scale [34]

^b^Scores for food security range from 0 to 8, with higher score (following transformation with raw scores subtracted from 8) indicating higher food security

### Quality-of-life (physical and mental health)

We measured quality-of-life using three questions from the Centers for Disease Control’s Health-Related Quality-of-Life—4 questions (Table 3) [16]. Technical documentation from the Health-Related Quality-of-Life —4 provides extensive explanation of the measure’s psychometric characteristics [35, 36]. We determined quality-of-life by averaging the number of days in the previous month for physical health not good, mental health not good, and days in which poor physical and mental health prevented the respondent from doing their usual activities. Thus, our measure provides an indicator of reduced quality-of-life, with a possible range of 0 to 31. For the set of three questions making up the quality-of-life measure, internal consistency reliability was 0.88 and 0.81 for the pre-pandemic and pandemic conditions, respectively, indicating good reliability for the measure. Quality-of-life was measured in pre- and pandemic conditions and averages were compared using a paired sample t-test [32]. The variable “quality-of-life change” is the result of subtracting the pre-pandemic score from the pandemic score.

**Table 3:** Items contributing to the quality-of-life indicators in adults ages 40–100 in the U.S

Question^a^	
Now thinking about your physical health, which includes physical illness and injury, for how many days during the past 30 days was your physical health not good?	
Now thinking about your mental health, which includes stress, depression, and problems with emotions, for how many days during the past 30 days was your mental health not good?	
During the past 30 days, for about how many days did poor physical or mental health keep you from doing your usual activities, such as self-care, work, or recreation?	

^a^Questions from the Centers for Disease Control’s Health-Related Quality-of-life—4 questions [16]

### Nutrition

We measured nutrition with the Dietary Screening Tool (DST) [37]. The 24-item short-form of the Dietary Screening Tool was developed and validated for use with middle-aged and older adults [38, 39]. The questions vary in number of response options and score points per option, resulting in a total possible range between 0 and 100 points. Healthier options within each question receive a higher score, so a higher total score indicates a more nutritious diet. Nutrition was measured in pre- and pandemic conditions and averages were compared using a paired sample t-test [32]. Internal consistency reliability for the DST was 0.61 and 0.62 (both *n* = 9969) for the pre-pandemic and pandemic conditions, respectively. The variable “nutrition change” is the result of subtracting the pre-pandemic score from the pandemic score.

### Statistical methods

Descriptive statistics were used to describe the sample (demographic variables and pre-existing health conditions). Planned comparisons using paired sample *t*-tests were used to test a priori hypotheses related to (1) changes in health-related variables in pre-pandemic and pandemic conditions (four tests), and (2) identical planned comparisons considered separately based on minority sample (yes or no) (eight tests). Correlations between a set of variables were examined in an exploratory fashion. Tests for statistical significance were based on α = 0.05, and appropriate effect sizes were also evaluated using Cohen’s standard levels of effect size [40].

## Results

### Demographic variables

We collected demographic data from participants across the four United States census regions: Northeast (*n* = 2966, 30% of total sample), Midwest (*n* = 2352, 24%), South (*n* = 2664, 27%), and West (*n* = 1987, 20%). The sample population was 57% female (*n* = 5733) and 43% male (*n* = 4236) and racially diverse (White *n* = 7330, 74% of total sample; Black *n* = 1381, 14%; Asian *n* = 694, 7%; Hispanic *n* = 536, 5%; Other Non-Hispanic *n* = 31, < 1%). Minority status was defined as “yes” if race was anything other than White (Non-Hispanic), so 26% of the sample was minority status.

Participants ages ranged from 40 to 100 years (*m* = 62.1, SD = 11.2): middle-aged (40 to 59, *n* = 3503, 35% of total sample), older (60 to 74, *n* = 5364, 54%), and oldest (75 to 100, *n* = 1102, 11%). Education level varied among the sample: less than high school (*n* = 124, 1% of total sample); high school/general equivalency degree (GED) (*n* = 1501, 15%); some college (*n* = 1848, 19%); associate or technical school degree (*n* = 1341, 14%), bachelor’s degree (*n* = 2845, 29%), graduate degree (*n* = 2295, 23%), prefer not to answer (*n* = 15, < 1%). Household income data were collected using a measure with eight levels: less than $10,000 (*n* = 363, 4% of total sample), $10,000 to $14,999 (*n* = 363, 4%), $15,000 to $24,999 (*n* = 811, 8%), $25,000 to $34,999 (*n* = 944, 10%), $35,000 to $49,999 (*n* = 1285, 13%), $50,000 to $74,999 (*n* = 1883, 19%), $75,000 to $99,999 (*n* = 1376, 14%), $100,000 and higher (*n* = 2537, 25%), and prefer not to answer (*n* = 407, 4%).

### Pre-existing health conditions

The health conditions reported by participants ranged from 0 to 14 (M = 2.23, SD = 1.94) from the full set of 20 possible health conditions. From the smaller set of six possible health conditions associated with more severe COVID-19 disease, the health conditions reported by participants ranged from 0 to 5 (*m* = 0.64, SD = 0.73).

### Changes in health-related variables pre- and pandemic

Table 4 reports a set of planned comparisons (using t-tests) between pre-pandemic and pandemic health and well-being variables, all of which were significant at the α = .05 level (Table 4). Because significance could have been due to the large sample size, so we calculated effect sizes using Cohen’s *d* [40]. Cohen’s *d* for physical activity reached the small effect size, which means that physical activity was higher in pre-pandemic conditions than in pandemic conditions. Effect size did not reach the small level among other comparisons (*d* ≤ 0.20), so changes observed in food security, nutrition, and quality-of-life are negligible.

**Table 4:** Changes in self-reported health variables pre-COVID-19 and during COVID-19 by adults ages 40–100 in the U.S

Health variable	Pandemic condition						Paired-sample *t* test		Effect size^b^	
	Pre-pandemic		Pandemic		Change					
	Mean	SD	Mean	SD	Mean	SD	*t*	Significant at α = 0.05?	*d*	Label
Physical activity	3.41	1.69	3.00	1.76	−0.41	1.18	34.8	Yes	0.35	Small
Food security	7.10	1.98	7.33	2.02	.023	1.32	17.4	Yes	−0.17	–
Nutrition	51.4	11.7	51.0	11.9	−0.45	6.08	7.45	Yes	0.08	–
Quality-of-life	2.91	6.34	3.34	6.33	0.43	5.04	−8.54	Yes	−0.09	–

*n* = 9969

^a^*df* = 9968; 2-tailed test. All tests would also have been significant at α = 0.05

^b^Cohen’s *d* using the common interpretive categories (small, medium, large) [40]

Additional planned comparison tests were repeated separately for participants with minority status or White (non-Hispanic) (see Table 5). Consistent with the results for the whole sample, minority and non-minority groups showed a decrease in level of physical activity during the pandemic compared to pre-pandemic. Additionally, the minority group increased food security. All other changes in health and well-being from pre-pandemic to pandemic conditions for the minority and non-minority group were negligible (Table 5).

**Table 5:** Changes in self-reported health variables pre-COVID-19 and during COVID-19 by minority and non-minority adults ages 40–100 in the U.S

Population	Health variable	Pandemic condition						Paired-sample *t* test		Effect size^b^	
		Pre-pandemic		Pandemic		Change					
		Mean	SD	Mean	SD	Mean	SD	*t*	Significant at α = 0.05?^a^	*d*	Label
Minority	Physical activity	3.48	1.63	3.02	1.73	−0.46	1.32	18.0	Yes	0.35	Small
	Food security	6.78	2.12	7.13	2.21	0.36	1.59	−11.7	Yes	−0.23	Small
	Nutrition	52.3	11.7	51.7	12.0	−0.57	7.00	4.16	Yes	0.08	–
	Quality-of-life	2.24	5.64	3.11	6.11	0.86	5.01	−8.85	Yes	−0.17	–
Non-minority White	Physical activity	3.38	1.71	2.99	1.78	−0.39	1.13	29.9	Yes	0.35	Small
	Food security	7.22	1.91	7.40	1.94	0.18	1.21	−12.9	Yes	−0.15	–
	Nutrition	51.1	11.7	50.7	11.9	−0.41	5.72	6.18	Yes	0.07	–
	Quality-of-life	3.15	6.56	3.43	6.41	0.28	5.04	−4.68	Yes	−0.06	–

^a^Minority *n* = 2629, *df* = 2638; non-minority *n* = 7330, *df* = 7329; 2-tailed test. All tests would also have been significant at α = 0.001

^b^Cohen’s *d* [40] using the common interpretive categories (small, medium, large)

### Exploration of correlations between variables

Table 6 reports the Pearson-*r* correlations between variables, including minority status (minority), level of education (education), income level (income), age level (age), and presence of at least one of 20 possible pre-existing health conditions (health). The variable also included physical activity, food security, nutrition, and quality-of-life as assessed using their associated “change” variables from pre-pandemic to pandemic conditions. Cases were dropped from analysis when participants elected to not respond to questions contributing to either education (*n* = 15) or income level (*n* = 407), resulting in approximately 4% of cases dropped, with subsequent *n* = 9552. None of the correlations reached significance at the 0.05 level (*N* = 1000). No correlations between change variables and the other variables reached the level of even a small effect size using Cohen’s [40] categories, regardless of their level of statistical significance. These analyses were exploratory and did not reflect a priori hypotheses.

**Table 6:** Pearson-*r* correlations between variables

	Minority	Education	Income	Age	Health	PAC	FSC	NC	QOLC
Minority	1								
Education	−0.06	1							
Income	−0.14	0.46	1						
Age	−0.30	0	0.03	1					
Health	−0.13	−0.07	−0.10	0.23	1				
PAC	−0.03	−0.01	− 0.01	0	− 0.01	1			
FSC	0.06	−0.02	−0.07	− 0.08	−0.02	0.04	1		
NC	−0.01	0	0.02	0.02	−0.01	0.07	0.09	1	
QOLC	0.05	−0.01	−0.05	− 0.13	0.05	− 0.06	−0.05	− 0.05	1

*n* = 9552

*Abbreviations: Minority* minority status, *Education* education level, *Income* income Level, *Age* age level, *Health* presence of 20 possible pre-existing health conditions, *PAC* Physical activity change, *FSC* Food security change, *NC* Nutrition change, *QOLC* Quality-of-life change

Pearson-*r* correlations of 0.062 or greater are significant (for *n* = 1000, with 2-tailed test) when α = 0.05, so none of the correlations in this table are significant

## Discussion

### Physical activity

Exercise and diet are two ways in which older adults can maintain a healthier lifestyle and help prevent illness. As predicted, our results suggest that physical activity is a health and wellness indicator that changed during the pandemic, with a decline in physical activity in adults ages 40 to 100. Similar findings confirmed a decrease in physical activity in some individuals in Italy, Canada, and Belgium. Physical activity, especially walking, decreased in undergraduate students in Italy, while sedentary behavior such as watching TV and computer use increased [41]. In Canada, 1098 adults over 19 years old were surveyed and individuals who were relatively inactive before the pandemic were more likely to become even less active during the strictest period of lockdown, whereas those who were relatively active before the pandemic were more likely to increase activity during lockdown [5]. Furthermore, inactive individuals who spent more time active outdoors had lower anxiety than individuals who spent less time being active outdoors [5]. Largely contrary to our results and those from Italy and Canada, adults in Belgium primarily increased their exercise level, but sedentary behaviors also increased and exercised decreased in some groups, especially those over the age of 55 years old [42].

A decline in physical activity could impact overall health of older adults, including their risk of severe complications from COVID-19. Older adults that participated in our survey had, on average, more than two of the 20 pre-existing health conditions we included on our survey. We recognized that many older adults are at risk of severe COVID-19 because of pre-existing health conditions; however, we noted that while physical activity decreased among many in our sample most were able to maintain some normalcy with respect to physical activity. New Zealand researchers showed similar finding in that many of the participants surveyed during March through May 2020 continued their physical activity routine [13].

Physical inactivity has been associated with reduced heart health, which increases the likelihood of sudden cardiac death, an increase in skeletal muscle decline, and a decrease in cognition [12]. In older adults, exercise reduces frailty (reduced capacity to reach physiological homeostasis) and sarcopenia (muscle atrophy, loss of muscle strengths and power); these conditions have negative outcomes following hospital discharges in older adults [12]. The World Health Organization and the United States Department of Health and Human Services [43, 44] recommend 150 min of moderate physical activity per week or 75 min of vigorous physical activity per week, while adding at least 2 days of muscle-strengthening exercises [6, 43–45]. Several authors suggested that some major public health initiatives in response to the pandemic — including “lockdowns” and social distancing protocols—have created personal, social, and environmental barriers to the ability of older adults to engage in physical activities [46, 47].

One group of researchers have argued that the imposition of such public health measures serves as a starting point for a vicious cycle of inactivity, muscle disuse, sarcopenia, and physical inactivity among older adults [9]. Our results support the idea that the pandemic may prevent older adults from engaging in physical activity. We found this effect regardless of minority status.

### Food security

We predicted that COVID-19 would exacerbate food insecurity because many local and state policies closed businesses, including food outlets. However, our results suggested otherwise, as participants reported a high level of food security. In fact, food security was somewhat increased among minority participants. We were puzzled by this finding as we predicted more food insecurity for older adults and minorities especially after the onset of the pandemic. Prior to COVID-19 many older adults (especially adults over 60) received meals through a variety of ways, including social services such as “meals on wheels” and “home meal delivery.” Perhaps food security increased because government-sponsored agencies increased meal delivery to this group after the onset of the pandemic. For example, Healthy New York and Seabury Home Meal Delivery services supported by the DC Department of Aging and Community Living ensured that older adults, especially those who were homebound, continued to receive healthy meals [48, 49]. Moreover, adults over 60 years and older in most urban cities have access to social services that provided meals and adults 40 to 59 years may have access to food from local food pantries. Organizations such as Feeding America have delivered an estimated 4.2 billion meals to people in the United States from March through October 2020 [50].

### Quality-of-life (physical and mental health)

Participants reported between two (2) and three (3) days in the prior month in which their physical and/or mental health was not good before the pandemic and this did not change during the pandemic. So, overall quality-of-life for participants remained relatively stable, indicating an underlying resilience in this population. Resilience is defined as the process of adapting well in the face of adversity, trauma, tragedy, threats, or significant source of threats [51]. The answers of older adults, regardless of minority status, suggests quality living and an ability to adapt to circumstances during the COVID-19 pandemic crisis, which demonstrates high level of resilience, especially since their physical activity was reduced [51].

### Nutrition

Adequate nutrition during the pandemic has an added benefit of boosting the immune system and improving resistance to malnutrition in older adults. We predicted that COVID-19 would lead to less nutritious food choices in older adults. In older adults, malnutrition and undernutrition facilitate the onset of sarcopenia, which is particularly problematic in individuals with health conditions such as cancer, heart disease, or chronic infection [52]. Nutritional screening for malnutrition has been recommended as a part of routine clinical care [53, 54]. Additionally, it has been recommended that older adults consume a high protein diet at each meal to protect the skeletal muscles from wasting, but especially in the presence of acute or chronic illnesses [53–55]. Our results showed that older adults continued to consume the same amounts of fruits, vegetables, and proteins before and since the onset of COVID-19. This was unexpected given that access to nutritious food may have been challenging for some during the pandemic. While our current study showed no differences in consumptions patterns before and since the pandemic, we cannot conclude whether a sufficiently nutritious diet is being consumed. Research supports that older adults need a diet high in fruits and vegetables with adequate proportions of protein to increase resistance to non-communicable diseases [56].

### Food security findings in ethnic and minority participants

We previously noted the unexpected finding that food security increased among the minority population, when we predicted a decline in food security. We believed that many minority participants would be considered essential workers, living in densely-populated cities with limited access to fresh produce, and so would rely on unhealthy food choices. Researchers had reported poor nutrition increased during the early phases of the pandemic, leading to a pronounced impact on minorities with a disproportionate burden of chronic diseases such as hypertension, diabetes, and asthma [57, 58]. We also recognize that minorities, especially African Americans, have a higher burden of chronic diseases and have been shown to have a higher death rate from COVID-19 than any other ethnic and minority group [59]. In fact, it has been reported that African Americans had a 3.4 times higher mortality rate than non-Hispanic Whites [59].

Our results may run counter to other studies that show a decline in food security among minorities because of the size of our sample, which is much larger than many studies, and was collected at a different timeframe from other studies. Early research on COVID-19 focused on March through June 2020 when lockdowns were the most restrictive in many states in the United States. Since we collected data in August and September 2020, the rates of COVID-19 were lower in the United States and restrictions eased, providing participants more mobility. Many minority respondents who were working, may have had access to food and those who were not working may have had access to soup kitchens and food pantries.

Additionally, in states like New York, there was a major push to increase enrollment in the Supplemental Nutrition Assistant Program (formerly known as “Food Stamps”, especially among older adults, and it is possible that other states across these regions made similar efforts [60]. In fact, the USDA initiated the SNAP online pilot, to which New York was one of the early adopters, that allowed SNAP benefits to be used online [61]. We suspect that many states-initiated safety nets like these to assure that many citizens were able to have enough healthy food during the pandemic. We also considered the possibility that since our data collection was later in the year, that local jurisdictions were the beneficiaries of learned lessons of what worked and what did not and were able to adjust implementation of their safety nets for their citizens more efficiently. We believe that because many older adults were homebound, it is possible that large proportion reconnected to cooking their own meals and/or sharing cooked meals with others. We suspect that food insecurity was less of an issue as spending on grocery took precedent over other types of spending such entertainment or traveling, which in many ways left citizens with the ability to save more money that was perhaps used to purchase healthy food. Lastly, while the survey aim was to target a representative sample of older adults across the four US census regions, it is possible that the respondents to this survey were more middle-income individuals and may have been in a better economic position to endure the COVID-19 storm.

## Conclusion

There is a growing body of research on the plight of older adults in the wake of COVID-19 and how it affects health conditions, physical activity, food security, quality-of-life, and nutrition. Our aim was to sample a large population of adults 40 years and older in the four U.S. census regions and to target urban cities and populations that were at high risk for suffering from severe COVID-19 complications. Populations we considered high risk include minorities, less educated, lower socio-economic status, older, and those with underlying health conditions. For example, members of minoritized groups have been reported to be at greater risk for contracting COVID-19 and experience worse health outcomes or greater rates of death than Whites, which aligns with our understanding of health disparities and health inequities when minorities are not able to access adequate and quality health care [59, 62]. Hypertension, diabetes, asthma, and other chronic diseases are prevalent in Black and Hispanic populations, resulting in an elevated risk factor for COVID-19. Blacks and Hispanics are disproportionately represented as essential workers without the luxury of working from home [63]. Many Blacks and Hispanics are more likely to live in densely populated housing, perhaps placing them at increased risk for transmission of the virus. Therefore, we intentionally targeted urban cities since minorities are likely to make up a greater percentage of the population in these areas, and thus would be represented in our study.

### Limitations and implications for practice

One limitation to our study was that despite our efforts to target minoritized individuals, Whites were most likely to respond to the survey. We report this as a limitation given our aims, but our sampling closely aligns with the demographics in the United States [64]. A second limitation was that the survey was offered online. It is possible that there was a technology bias that skewed results toward those who could afford and/or access a computer. The survey could be completed on a smart phone, which we anticipated would have mitigated some of the technology bias in our sampling, but this technology is also not ubiquitous. A third limitation was that onset and effects of COVID-19 were not similar throughout the country, nor were the responses by local and state governments. Our survey gives a snapshot of what conditions were like during the first peak of the pandemic in the U.S., but conditions became worse. A fourth limitation was that the reliability of the physical activity scale and DST were lower than desired, so a suggestion for future research would be to create a tool with greater reliability.

It is concerning that physical activity declined because this helps maintain physical and mental health and is also an important time to socialize for many older adults. In many ways our data indicate that the older adult population in U.S. cities may be more resilient than expected during the pandemic. However, the pandemic could have negative impacts that we did not detect, either due to the survey instrument or the timing of our survey.

### Recommendations

Our recommendation for future research should look more closely at the impacts of COVID-19 on high-risk groups, including minorities. For example, an analysis of the minority population, separated by regions of the U.S., in relation to physical activity, nutritional status related to food intake, and quality-of-life indicators based on differential age categories is warranted. Given the evolving nature of the pandemic and the high risk of individuals we studied, health and well-being of older adults should continue to be monitored so impacts can be mitigated.

We recommend developing of interventions that target minority older adults and increase awareness of the benefits of physical activity on physical and mental well-being in times of crisis that are especially beneficial to increase resilience in this population. There has been some recent research that highlights the benefits and barriers of physical activity and exercises in older adults [65]. We know that increasing physical activity improves health outcomes, lowers the burden of chronic diseases, and improves the overall quality-of-life for all people to include people in racial and ethnic minority groups. Further, community engagement of ethnic racial minority groups must be embedded in any public health response that seeks to decrease the disparate health treatment suffered by minorities in the current health care system [66].

## Data Availability

The datasets used and/or analyzed during the current study are available from the corresponding author on reasonable request.
